# Alcohol, myocardial infarction and survival: published estimates require correction and reanalysis

**DOI:** 10.1080/02813432.2026.2728001

**Published:** 2026-09-08

**Authors:** George Koulaouzidis, Anastasios Koulaouzidis

**Affiliations:** a Department of Biochemical Sciences, Pomeranian Medical University in Szczecin, Szczecin, Poland; b Department of Gastroenterology, Pomeranian Medical University in Szczecin, Szczecin, Poland; c Surgical Department A, Odense University Hospital, Odense, Denmark; d Department of Clinical Research, University of Southern Denmark, Odense, Denmark

**Keywords:** Alcohol consumption, myocardial infarction, all-cause mortality, survival analysis, competing risks, epidemiology

## Abstract

**Objective:** To assess the internal consistency and interpretation of estimates reported in a 30-year cohort study of alcohol consumption and cardiovascular outcomes. **Design:** Critical commentary with independent recalculation of odds ratios from published aggregate data. **Setting:** A Swedish occupational cohort study with 30 years of follow-up. **Subjects:** Published data for 973 male automotive workers. **Main outcome measures:** Myocardial infarction, stroke, all-cause mortality and survival-time estimates. **Results:** The published abstract attributes an odds ratio of 0.57 to myocardial infarction, whereas the reported counts yield an odds ratio of 1.41; the 0.57 estimate relates to all-cause mortality. Further concerns include survival-time terminology, numerical inconsistencies across tables and figures, competing-risk methods, multiplicity and residual confounding, including sick-quitter bias. **Conclusion:** The published record requires correction and reconciliation with the underlying data and statistical analyses. The findings support only exploratory associations and do not establish cardioprotection or a survival benefit from alcohol consumption.

Asp and Dimberg’s 30-year follow-up of Swedish male automotive workers addresses an important clinical question [1]. However, the published report contains a central result-transfer error and several numerical and analytical inconsistencies that warrant correction and clarification.

The most consequential problem is in the abstract. It states that alcohol consumption was associated with lower odds of myocardial infarction (MI), reporting OR 0.57 (95% CI 0.41–0.79). Table 5 instead reports 81 MIs among 737 alcohol consumers and 19 among 236 non-consumers, corresponding to OR 1.41 (95% CI 0.84–2.40; *p* = 0.197). The OR of 0.57 belongs to all-cause mortality: 160/737 deaths among consumers versus 77/236 among non-consumers. The mortality estimate has therefore been assigned to the wrong outcome in the abstract, reversing the direction of the headline MI comparison. This warrants prompt correction.

The reported ‘median survival advantage of 1.3 years’ is also not supported by the presented data. Only 237 of 973 participants died, and mortality was below 50% in both alcohol-consumption groups; median survival was therefore not reached. Table 12 reports Kaplan–Meier estimated mean times of 26.8 and 28.1 years. These values may represent the area under the Kaplan–Meier survival curve truncated at the observed follow-up horizon; if the authors intended restricted mean survival time (RMST), the truncation time and between-group difference with 95% CI should be reported explicitly. They cannot be interpreted as median survival or unrestricted life expectancy [2].

Further internal discrepancies merit clarification. In Table 2, the three exercise categories total 981 participants, while Figure 4 shows 304, 487 and 186 participants (total 977); neither matches the cohort size of 973. In Table 3, the reported median total alcohol intake for the 55–84 g/week category is 40 g/week and for the 85–342 g/week category is 67 g/week, values outside their respective category ranges. In Table 6, the wine–stroke counts are 41/451 versus 48/521; a standard 2 × 2 calculation gives OR 0.985 (95% CI 0.636–1.526; Pearson *p* = 0.948), not the reported OR 0.87 (95% CI 0.64–1.20; *p* = 0.380). Tables 11 and 13 contain identical MI Kaplan–Meier results. The Methods also assign 6 g ethanol to a beer ‘drink’, whereas Figure 2 defines one standard drink as 12 g. Without the original questionnaire and coding this does not establish an exposure error, but it requires clarification. These points would benefit from reconciliation with the source data and original statistical output.

The inferential framework also deserves caution. Alcohol exposure was self-reported at baseline, whereas outcomes accrued over three decades. Although the Introduction acknowledges the ‘sick-quitter’ problem, the study does not report whether its own non-consumer group comprises lifetime abstainers, former drinkers or illness-related quitters. Drinkers and non-drinkers also differed markedly in occupational and marital status: 69% versus 36% were clerical workers and 79% versus 69% were married/cohabiting. These selection effects, socioeconomic differences, residual confounding and possible reverse-causation or sick-quitter bias substantially limit causal interpretation of the observed survival association. Contemporary meta-analytic and Mendelian-randomisation evidence likewise cautions against inferring cardioprotection from low-volume alcohol use [4,5].

For MI and stroke, death from other causes is a competing event. The Methods state that Kaplan–Meier cumulative-incidence analyses censored participants at death. If the target is observed cumulative incidence of MI or stroke, treating competing deaths as simple censoring can overestimate event probability; a competing-risk cumulative-incidence estimator is preferable, while cause-specific or subdistribution hazard models should be selected according to the estimand [3]. The adjusted associations for total alcohol and mortality (*p* = 0.045) and beer and MI (*p* = 0.049) also arise amid numerous outcome, beverage, exposure-category and subgroup comparisons without a stated primary contrast or multiplicity strategy. These findings should therefore be regarded as exploratory. The linear per-gram Cox models additionally require demonstration of functional form and proportional-hazards assumptions.

We therefore consider the study to support, at most, exploratory associations between baseline reported alcohol intake and later outcomes in a selected occupational cohort. It cannot establish an alcohol-related survival benefit or support reconsideration of public-health advice. We respectfully invite the authors to correct the abstract and indexed record, clarify the survival estimate and terminology, reconcile the relevant tables, and consider verification of the principal analyses against the underlying data and statistical code as part of the correction process. Pending that clarification, the principal conclusions should be interpreted with caution.

## Data Availability

No new datasets were generated. The numerical checks in this correspondence are based on aggregate data reported in the published article and the cited literature.
